# Emerging Noninvasive Biomarkers, and Medical Management Strategies for Alcoholic Hepatitis: Present Understanding and Scope

**DOI:** 10.3390/cells9030524

**Published:** 2020-02-25

**Authors:** Khushboo S. Gala, Vatsalya Vatsalya

**Affiliations:** 1 Division of Internal Medicine, University of Louisville, Louisville, KY 40202; USA; 2 Division of Gastroenterology, Hepatology, and Nutrition, University of Louisville, Louisville, KY 40202, USA; 3 Robley Rex VA Medical Center, Louisville, KY 40292, USA

**Keywords:** alcohol, novel biomarkers, medical management, alcoholic hepatitis, noninvasive biomarkers, alcoholic liver disease

## Abstract

Alcohol use disorder is associated with a wide array of hepatic pathologies ranging from steatosis to alcoholic-related cirrhosis (AC), alcoholic hepatitis (AH), or hepatocellular carcinoma (HCC). Biomarkers are categorized into two main categories: biomarkers associated with alcohol consumption and biomarkers of alcoholic liver disease (ALD). No ideal biomarker has been identified to quantify the degree of hepatocyte death or severity of AH, even though numerous biomarkers have been associated with AH. This review provides information of some of the novel and latest biomarkers that are being investigated and have shown a substantial association with the degree and severity of liver injury and inflammation. Importantly, they can be measured noninvasively. In this manuscript, we consolidate the present understanding and prospects of these biomarkers; and their application in assessing the severity and progression of the alcoholic liver disease (ALD). We also review current and upcoming management options for AH.

## 1. Introduction

Alcohol consumption has a major role in the development of alcoholic liver disease (ALD) in the United States [1]. Cirrhosis is the 12th leading cause of death in the United States [2]. The phenotypical manifestation of ALD consists of steatosis, steatohepatitis, alcoholic cirrhosis (AC), alcoholic hepatitis (AH) and hepatocellular carcinoma (HCC) depending on the chronic and heavy nature of alcohol intake [3,4,5,6]. Only one-third of the heavy drinkers develop any form of liver damage with only 10–15% of all drinkers develop severe forms of ALD [7]. This has resulted in identifying several factors and pathologies that alter the risk of developing advanced form of ALD especially AH [8].

The metabolism of alcohol results in the buildup of pro-inflammatory biochemicals resulting in a wide array of pathological changes in the liver through direct cytotoxic metabolites, activation of proinflammatory pathways, and fatty acid oxidation [9]. Several biomarkers have been identified and have been linked as a possible therapeutic target to reduce inflammatory pathways in the liver [10]. This has led to the classification of biomarkers based on both alcohol consumption as well as ALD. Established markers of liver damage (AST and ALT) are highly nonspecific [11], and are affected by a wide array of pathologies. This creates a gap in understanding and characterization of AH as well, which has been historically classified using surrogate biomarkers. Few scores have been validated to determine treatment strategies and prognosis in AH, however their efficacy has been limited. Newly tested novel biomarkers could determine the degree of liver inflammation, prognosis and may be used in deciding the appropriate treatment.

The medical management of AH has observed several positive changes in the last few decades. Comprehensive and targeted treatment options are limited, however emerging research on the responses of proinflammatory cytokines, role of hepatocyte death, and expanded understanding of the gut–liver axis could present novel alternatives. The aim of this review is to provide new insights on the pro-inflammatory and hepatocyte death biomarkers associated with AH, their usefulness as noninvasive methods of investigation, and possible therapeutic and prognostic applications. In brief, we have also provided review of the current and upcoming therapeutic options for AH.

## 2. Biomarkers

### 2.1. Static Biomarkers

The current diagnosis of AH [12] is based on the history of alcohol consumption, physical examination and laboratory finding (AST, ALT and GGT). However, these diagnostic criteria vary significantly in patients and may not accurately predict the degree of liver inflammation. In the last couple of decades, new research has led to the development of several novel clinical criteria scores, and biomarkers that showed better efficacy in associating with the severity of AH [13]. They may provide a greater diagnostic, prognostic, and possible therapeutic options when combined with current guidelines for AH.

“Static” scores or models are used to determine prognosis or treatment modalities based on variables obtained from a single point in time. Scores like the Discriminant Function Index (DF); Glasgow Alcoholic Hepatitis Score (GAHS); the Age, Bilirubin, INR, and Creatinine (ABIC) score; and the Model of End-Stage Liver Disease (MELD) score have been validated, and currently being used in clinical practice.

#### 2.1.1. Discriminant Function (DF)

The Discriminant Function Index (DF, also known as Maddrey’ s DF Score) is one of the most widely used and validated scores that was developed to evaluate the benefit of corticosteroid therapy in patients with AH, and application of diagnostic targets [14,15]. It uses Prothrombin Time (PT) and its higher normal range value, and total bilirubin as variables; and a DF ≥ 32 has been used to define severe AH [16,17]. Studies have shown that patients with DF ≥ 32 and no treatment have a mortality rate of 20–50% over 30 days; depending on practices of supportive care [18,19]. Current American College of Gastroenterology guidelines recommend treating AH patients with corticosteroids who have DF ≥ 32 [16,20]. Limitation of this score includes non-standardization of the PT values, and different upper value of the normal range as a laboratory variation depending on the type of thromboplastin used.

#### 2.1.2. Model of End-Stage Liver Disease (MELD)

The Model of End-Stage Liver Disease (MELD) score was originally developed to predict survival in patients with cirrhosis undergoing transjugular intrahepatic portosystemic shunting [21]. Over the years, it has been used to predict survival for patients with cirrhosis; and is the basis for organ donor allocation in liver transplantation [22]. It has been validated and now increasingly used in severe AH patients for predicting 30-day and 90-day mortality [23,24]. The original MELD score uses bilirubin, creatinine, INR (a new derivation MELDNa incorporates serum sodium) as variables. A MELD score of over 20 defines severe AH with an approximate 20% mortality [25]. Some of the advantages of the MELD score over the DF are the use of INR as compared to prothrombin time, and the use of serum creatinine to include renal function, which plays an important role in the prognosis of AH patients [16]. Several studies have estimated that MELD may be equivalent to or even better than the DF as a prognostic marker of AH [26,27,28].

#### 2.1.3. Age, Bilirubin, INR and Creatinine (ABIC) Score

The Age, Bilirubin, INR and Creatinine (ABIC) score served as a simple tool for 90-day and one year mortality risk stratification in the AH patients [29,30]. AH patients are classified as low (<6.71), intermediate (6.71–9) and high (>9) risk for 90-day and one-year mortality (100%, 70% and 25% of survival rate respectively). This score is well-validated; however, a limitation is that the negative predictive value for mortality is significantly better than the positive predictive value [18].

#### 2.1.4. Glasgow Alcoholic Hepatitis Score (GAHS)

The GAHS model has been developed to predict mortality with the help of an optimal cutoff level [31,32]. It uses age, serum bilirubin, blood urea nitrogen, PT ratio and the peripheral white cell count as variables, with a score of <9 being associated with an extremely poor mortality [33]. Research has shown that GAHS ≥ 9 may benefit from corticosteroid treatment, whereas those with a GAHS < 9 are unlikely to benefit from such treatment even if the DF is ≥32 [34,35]. One report suggests that ABIC, GAHS and MELD could be superior to the DF score [33].

However, none of these markers use the degree and severity of liver cell death; and have not been fully efficient in characterizing the diagnostic or prognostic aspect of AH. Thus, larger clinical and mechanistic studies are needed to compare these static models and provide evidence-based development of markers that could correspond well with the liver Injury.

## 3. Novel Emerging Biomarkers

Direct biomarkers are related to alcohol consumption and the various pathologic alterations in human metabolism. These biomarkers are associated with the direct cytotoxic metabolites produced by alcohol metabolism that could promote inflammation of the liver. These newly identified biomarkers can be used to estimate the degree of alcohol intake and alcohol-induced liver injury by noninvasive methods. Although these biomarkers have an established association with the disease process and severity, there has yet to be any standardization of results and the clinical applications have yet to be determined. It is not in the scope of this review to accommodate and address all the emerging biomarkers. This review addresses a few selected ones that have gained interest in AH studies (Table 1).

### 3.1. Biomarkers of Liver Cell Death and Regeneration

Hepatocellular death is a hallmark of ALD, and its magnitude correlates with the severity of disease [59]. Some molecules have been found to be indicative of liver injury, and may serve as biomarkers for the diagnosis and prognosis of ALD. These are described below.

#### 3.1.1. Cytokeratin 18

Cytokeratins are a group of intracellular intermediate filament protein in cells that have multiple functions including intracellular vesicle support, regulate cell cycle progression, cell division and maintains cellular integrity [60]. The hepatocyte cytoskeleton consists mainly of cytokeratin 8 and 18 [61]. Hepatocyte stress/inflammation disrupts the cytoskeleton resulting in hepatocyte ballooning and Mallory body formation, both of which are a hallmark of AH. These histological findings are nonspecific and are caused by numerous pathologies including ALD, NASH and cholestasis [62]. Cytokeratin filaments are released during hepatocyte damage and are associated with the degree of liver inflammation [63].

Cytokeratin 18 (K18 or keratin 18) is the most abundant intermediate filament component that is readily detectable in hepatocyte damage. It is broken down into two forms via caspases, K18M65 and K18M30, both of which are also detected in plasma via ELISA [64]. In one study determining the efficacy of K18 in AH, serum K18 levels were more indicative of the degree of liver injury than AST and ALT and may eventually be used to predict prognosis in alcoholic liver disease [11,36]. This study discussed about the usefulness of K18M65/ALT as a clinical determinant for differential diagnosis of AH comparing with NASH [36]. Bissonnette et al. [37] compared plasma level of M65 and M30 to histologically confirmed cases of AH with significantly positive results including a positive predictive value of 91%, the negative predictive value of 88% and a receiver operating characteristics curve to be 0.84 ensuring diagnostic accuracy. Its role in therapeutic efficacy is yet to be determined.

#### 3.1.2. Augmenter of Liver Regeneration

Augmenter of Liver Regeneration (ALR) is a protein that promotes liver regeneration, originally identified from the rat livers [65]. It has various functions, including inducing protein Fe/S maturation, sulfhydryl oxidase enzymatic activities and mitochondrial homeostasis [66]. Loss of ALR from mitochondria causes depletion of ATP, leading to the death of hepatocytes. ALR levels are increased when hepatic tissues are subjected to insults, indicating that it may be a damage responsive protein [39]. Studies on animal models have shown that decreased ALR in hepatocytes induces upregulation of microRNA that is responsible for reduced peroxisomal integrity and function (specifically, miR-540) [38]. In animal models with advanced ALD, ALR is reduced in liver tissues [67]. With future translational research in humans, ALR may serve as an important marker of ALD specially AH; however, such studies have not been reported yet.

### 3.2. Biomarkers of Immune Response

Recent clinical and experimental research has shown that both innate and adaptive immunity plays a large role in the pathogenesis of ALD [68]. Alcohol activates innate immunity; whereas oxidative modification of hepatic constituents induces adaptive immunity [69]. Immune responses trigger the inflammation and drive the progression of ALD. The molecules listed below are emerging biomarkers of immune response.

#### 3.2.1. CD 163

Cluster of differentiation (CD) is a classification system for surface proteins expressed on leukocytes utilizing immunofluorescent antibodies that bind to specific proteins [70]. Surface proteins have numerous functions including acting as a receptor, transport channel, enzyme activity and intracellular identification and interaction. In the liver, CD glycoproteins are mainly detected on Kupffer cells [28]. Some markers like CD163 are also released in response to the bacterial stressors like lipopolysaccharides (LPS), which are detectable in the plasma making this marker relatively easy to measure [71].

CD163 is a macrophage receptor protein on Kupffer cells sensitive to both Gram-positive and negative bacteria as well as hemoglobin–haptoglobin complexes [72]. It is hypothesized that alcohol metabolism results in multiple inflammatory cascades and toxic metabolites, including LPS, reactive oxide species (ROS), acrolein, etc. resulting in Kupffer cell proliferation and soluble CD163 (sCD163) shedding to limit damage to the liver [73]. Role of CD163 in portal hypertension and survival in AC has also been reported in one study [74]. Plasma CD163 can be measured using ELISA kit (RayBiotech Inc., Norcross, GA). In one study [41], sCD163 levels were 30% higher in AH when compared alcoholic cirrhosis and 10 times higher when compared to a healthy control group. Another study compared sCD163 to the degree of liver inflammation, which reported severely elevated sCD163 levels were associated with disease severity of liver and its poor outcomes [40]. In conclusion, sCD163 has a diagnostic, prognostic value and a possible therapeutic function in AH that needs further investigation.

#### 3.2.2. ST2 Receptor

The ST2 receptor encapsulates three forms of protein receptors, which are a plasma soluble isoform of ST2, a transmembrane isoform and an undetermined localization isoform [75]. While the plasma soluble receptors have shown a significant relation to the liver inflammation, the transmembrane form on Th2 cells has been linked to hepatic fibrosis. IL33 binds to the ST2 receptor, which activates the Il1R accessory protein pathway [76]. This initiates an inflammatory cascade resulting in the hepatic inflammation and fibrosis. Il33 also functions in promoting TH2 cell proliferation and migration to the liver, exacerbating the damage induced by the reactive alcohol metabolites [77]. The ratio of IL-33/sST2 could be an useful biomarker in the staging of disease severity of ALD [77].

Several studies have linked plasma levels of ST2 receptors to liver inflammation (involved in multiple pathological pathways) including AH [42]. Plasma levels of plasma soluble ST2 receptors can be detected and measured using ELISA [78]. It is worth mentioning that IL-33 and ST2 complex results in both proinflammatory and anti-inflammatory pathways, and levels may be associated with the either path, depending on the duration of insult [79]. The significance of the ST2 receptor is yet to be determined but remains as a possible diagnostic factor to determine the degree of inflammation and severity of liver and a potential target for therapeutic intervention [43].

#### 3.2.3. TNF- Related Apoptosis-Inducing Ligand (TRAIL)

Tumor necrosis factor is an inflammatory cytokine associated with acute inflammation. In the liver, it is released by the Kupffer cells activation. Numerous mediators such as LPS, ROS and direct alcohol-related toxicity lead to liver injury. Natural killer (NK) cells intervene in hepatic fibrogenesis by upregulating TRAIL and INF-gamma (anti-inflammatory cytokines), which is adversely affected by alcohol exposure that leads to the decreased effects of NK cells, allowing a upregulated pro-inflammatory response [80]. In one study using wild-type mice as a study model, mean serum ALT was three times higher than the TNF-α receptor 1 (TNF-R1) knockout mice on an alcohol-fed diet emphasizing the importance of TNF-α in alcohol-related liver injury [81]. TNF- related apoptosis-inducing ligand (TRAIL) is a cytokine that mediates in anti-inflammatory pathways by inducing apoptosis by activating caspase pathway [44]. One preclinical study showed that PEGylated TRAIL treatment ameliorates ALD in rats by eliminating activated hepatic stellate cells [82]. TRAIL activation alone in healthy liver likely does not develop apoptosis signifying the importance of pathological stressors (alcohol metabolite or viral infection). The combination of alcohol, pro-inflammatory metabolites and TRAIL activation (along with other cytokines’ activation) activated DR4 and DR5 (which contain death domains) likely results in the activation of the apoptotic pathway [45]. Neutrophilia and neutrophilic invasion of the liver in AH promotes proinflammatory pathways in the liver by secreting chemokines and cytokines. The severity of AH has been found to be inversely related to serum levels of TRAIL [46], which can be measured by a Western blot test. TRAIL is a potential marker to determine the severity of AH, and a possible therapeutic target in AH.

#### 3.2.4. Immunoglobulins (IgM, IgG and IgA)

Individuals with ALD are at a higher risk of infection due to a compromised immune system [83]. Research has suggested coinfection with *Polymorphous gingivalis* (*P. gingivalis*) may potentiate the effects of alcohol-related liver disease [84]. Significantly higher plasma anti-*P. gingivalis* IgG and IgA, and Anti-*P. gingivalis* W83 IgM were observed in ALD, especially with the diagnosis of severe AH [47].

#### 3.2.5. MicroRNAs (MiR-155 and MiR- 223)

MicroRNAs (miRNAs) are small (less than 50 nucleotides) noncoding RNAs that regulate the expression of their respective target messenger RNA (mRNAs), and encoded proteins at the posttranscriptional level [85]. Within the liver, miRNAs influence a wide array of critical biological processes including hepatocyte regeneration, metabolism, immunity, bile secretion, fibrosis and hepatocellular carcinoma (HCC) [86]. In addition to being housed intracellularly, miRNAs can also be detected extracellularly in the serum, plasma and other body fluids (saliva, urine). The high stability and easy detection of miRNAs in the circulation make them attractive as a potential biomarker for the liver diseases [48].

MiR-155 is a multifunctional miRNA located within the third exon on chromosome 21. A recent study [87] found that miR-155 deficiency attenuates chronic alcohol-induced steatosis, oxidative stress, and liver injury in the liver. The study showed that alcohol produces both M1 (classical activation) and M2 (alternatively activated profibrotic) macrophage activation in mice. In addition to macrophage activation, AH is also characterized by neutrophil infiltration to the liver. Neutrophil infiltration has been shown to correlate with the severity of acute AH [88]. Alcohol diet also resulted in an infiltration of neutrophils (CD11b + Ly6Ghi) in the livers of wild type mice. However, neutrophil infiltration was prevented in miR-155 KO mice after alcohol-fed diet. Collectively, miR-155 seems to play a promoting role in the occurrence and development of ALD.

MiR-223 (encoded on chromosome 12) is one of the most abundant miRNAs in the neutrophils. Previous studies [89] have shown that the upregulation of miR-223 plays a crucial role in terminating the acute neutrophilic response and could be a therapeutic target for the treatment of acetaminophen (APAP) induced liver failure. One study showed that alcoholics had elevated serum miR-223 levels compared with healthy controls [49]. In addition, in a chronic-plus-binge alcohol-fed mouse model, the levels of miR-223 were increased both in the serum and the neutrophils. However, another study found that the serum miR-223 levels increased while miR-223 levels in the neutrophils decreased in human alcoholics [50]. Another recent study showed that microRNA122 regulated by GRLH2 protects livers of mice and patients from ALD [90]. These discrepant findings suggested that the levels of hepatic neutrophils might be a critical factor for determining the outcome of potential therapeutic implications of miR-223 for diagnosing/treating ALD, making it more sensitive in patients with AH as compared to other manifestations of ALD.

### 3.3. Biomarkers of Metabolic Changes

Alcoholic liver disease leads to a range of metabolic disturbances, some of which can be assessed to determine the severity and prognosis of the hepatocellular damage [91]. A few such biomarkers, which have been gaining interest in ALD are listed below.

#### 3.3.1. Stearoyl-CoA Desaturase 1 (SCD1)

One of the histopathological findings in alcohol-induced liver injury is steatosis. This has been attributed to alcohol consumption that alters several metabolic processes, especially fatty acid metabolism leading to steatohepatitis. Alcohol is broken down via alcohol dehydrogenase to acetaldehyde, which thereafter is converted to acetate by acetaldehyde dehydrogenase altering the NADH/NAD ratio [92]. This imbalance of NADH to NAD results in the diversion of acetyl CoA toward ketogenesis and fatty acid synthesis [93]. Another mechanism that could contribute to the steatosis is the augmented response of sterol regulatory element-binding protein 1 (SREBP-1) in the presence of alcohol that results in increased fatty acid synthesis [94]. Stearoyl-CoA desaturase 1 (SCD1) is a rate-limiting enzyme that catalyzes the formation of monounsaturated fatty acids and reduced lipid synthesis. Promotion of the synthesis of monounsaturated fatty acids could play an important role in the development of steatosis and liver injury with chronic-plus-binge alcohol exposure [95]. Study on SCD1 knockout mice showed significant resistance to alcohol-induced hepatic inflammation in comparison to the control group, emphasizing the critical role of SCD1 in AH [52]. SCD1 activity can be measured indirectly by the palmitoleic acid to palmitic acid ratio via serum lipid measurements [52,96]. SCD1 remains as a possible therapeutic target that needs more clinical and translational investigations to elucidate its role in inhibiting the hepatic inflammation.

#### 3.3.2. Magnesium

Heavy alcohol drinking causes electrolyte and mineral disturbances, hypomagnesemia observed as one of the most common ones [97]. Acute alcohol abuse, chronic and heavy alcohol drinking could lead to hypomagnesemia [98,99,100]. Hypomagnesemia could occur in patients with hepatic steatosis originating from alcoholic and non-alcoholic liver disease [101]. Studies have shown that the degree of magnesium depletion found on muscle biopsy correlated with the increasing severity of liver injury in AC [102]. Heavy drinking marker heavy drinking days (HDD90) is the most important drinking pattern consequential in the development of subthreshold hypomagnesemia in early-stage ALD [54]. Overall, hypomagnesemia could be observed in the entire spectrum of AUD and liver disease [53]. Hypomagnesemia may serve as a risk factor [103] and marker [104] for ALD and could be useful in staging the severity of ALD.

#### 3.3.3. Uric Acid

Uric acid is a breakdown product of purine metabolism in humans. Elevated uric acid has been studied with respect to AUD, and alcoholic and non-alcoholic liver disease. Hyperuricemia has been described in heavy drinking [55,105,106], which can be attributed to downregulation catabolism of adenosine monophosphate [107] in heavy drinkers leading to the synthesis of uric acid as one of the major pathways involved. Several studies have described hyperuricemia in both ultrasound-diagnosed and biopsy-diagnosed non-alcoholic fatty liver disease as well [108,109]. In ALD, uric acid and ATP mediate an inflammatory cross-talk between damaged hepatocytes and immune cells and induce expression of LPS-inducible cytokines, IL-1β and TNF-α, by immune cells [106]. Hyperuricemia could be associated with liver cell death, gut barrier dysfunction and proinflammatory response in early ALD [55]. Emerging data shows that hyperuricemia serves an important marker for the pro-inflammatory response in ALD, and that appropriate treatment of ALD results in alleviation of hyperuricemia.

### 3.4. Biomarkers of Chemical Causes

The breaking down of alcohol releases highly toxic chemicals, which can trigger proinflammatory response consequential in the liver cell death [110]. The liver damage mediated by the pathological derivatives (free radicals, and highly reactive molecular fragments) can be synthesized by the dysregulated metabolic pathways due to the active participation of alcohol [57]. The damage is primarily directed towards the disruption of the essential components of cellular and intracellular membrane and their integrity [111,112]. Some of the emerging chemicals can be used as biomarkers of pathology with the findings published so far and are discussed below.

#### 3.4.1. Acrolein

Alcohol metabolism results in the production of highly toxic products including acetaldehyde, acetate, ROS and acrolein [113]. Acrolein is a toxic metabolite that is produced under physiological conditions but is quickly removed via natural scavengers in the body including β-alanyl histidine [114]. Acrolein has been shown to increase intestinal permeability by direct cytotoxic effects and by downregulating of tight junctions. Acrolein causes endoplasmic stress by forming adducts with proteins, impairing the function and altering morphological integrity. Its catabolism, both hepatic and nephrotic, produces 3 hydroxypropyl mercapturic acid (3HMP). 3HMP can be detected in urine via tandem mass spectrometry [114]. Acrolein is a modified polyunsaturated fatty acid that has a well-documented association with alcohol metabolism [56]. These reactive products interact adversely with the pro-inflammatory mediators on the hepatocytes resulting in inflammation, injury and disruption of functions [57]. Acrolein has gained recent importance as a mediator in the development of ALD [115]. Role in diagnosis, prognosis and therapeutic efficacy is unclear.

#### 3.4.2. Resolvins

Resolvins are lipid mediators derived from omega-3 polyunsaturated fatty acids (•3-PUFAs) like docosahexaenoic acid (DHA), *n*-3 docosapentaenoic acid (n-3 DPA) and eicosapentaenoic acid (EPA) that counter-regulate proinflammatory responses [116]. There are at least ten recognized resolvin molecules that perform a variety of functions, including promoting resolution via uptake of debris by monocytes and macrophages, promoting apoptosis and enhancing bacterial killing/clearance [117]. Animal model have shown that alcohol-fed mice have altered levels of resolvins [118] along with the changes in EPA and DHA levels. Use of DHA and EPA in improving liver injury in non-alcoholic fatty liver disease (NAFLD) is already being studied [119]; future research may show the benefits in ALD. Preliminary data has shown improvement in liver injury by supplementation of resolvin D1 to mice [58,120]. The potential benefits of specific PUFA-CYP-derived metabolites (PUFA epoxides) can also be investigated as therapeutic target of ALD [121]. As with NAFLD [122], plasma and tissue levels of •3-PUFAs and resolvins can be used as a predictor of pathology; and diagnostic biomarker of ALD.

## 4. Management of Alcoholic Liver Disease

ALD is a multifaceted disease that has high morbidity and mortality in its advanced form. Early stage ALD is reversible with abstinence from alcohol, however this reversibility decreases with the advanced form of disease. Available medical management of ALD includes abstinence from alcohol, nutritional therapy, pharmacological therapies and liver transplantation (Table 2).

### 4.1. Alcohol Abstinence

Abstinence from alcohol is the mainstay of management of ALD [123]. Three medications have been approved by FDA for AUD treatment: Disulfiram, Acamprosate and Naltrexone [124]. Only baclofen is approved for use in ALD [125,126]. The most effective strategy in management of AUD in ALD is a combination of psychosocial interventions, pharmacological therapy and medical management [127,128].

### 4.2. Nutritional Management

Patients with ALD often have micro- and macronutrient deficiencies [129,130]. Protein calorie malnutrition has been shown to have increased 30-day, 6-month and 12-month mortality rates; along with decreased risk of encephalopathy and infection [131,132]. A recent meta-analysis of 15 trials of enteral and parenteral nutrition found a 20% reduction in mortality in AH [133]. As per the American College of Gastroenterology practice guidelines, patients with severe AH need daily protein intake of 1.2–1.5 g/kg and caloric intake of 35 Kcal/kg, along with micronutrient supplementation (discussed later) [16].

### 4.3. Pharmacological Therapy

Little progress has been made so far on the development of pharmacological treatment of ALD, and there are currently no US Food and Drug Administration (FDA)-approved therapies for advanced ALD [134,135]. Many therapies have failed to showed efficacy, including *Silybum marianum* (milk thistle), propylthiouracil, colchicine, S-Adenosyl-L-methionine (SAMe) and polyenyl-phosphatidylcholine [136]. Some, currently used, other important and upcoming therapies [137] are highlighted further.

#### 4.3.1. Corticosteroids

Currently, the American Association for the Study of Liver Diseases and the American College of Gastroenterology practice guidelines recommend the use of corticosteroids for patients with severe AH [16,138,139]. Multiple trials performed on the efficacy of corticosteroids have showed mixed results. The largest trial performed (the STeroids Or Pentoxifylline for Alcoholic Hepatitis [STOPAH] study) showed only a trend for mortality benefit at 28 days with prednisolone, compared with patients receiving placebo (13.8% vs. 18%, *p* = 0.056) [140]. Recently, a meta-analysis examining 15 trials and greater than 1800 patients found weak evidence for no difference in all-cause mortality and serious adverse events between groups of corticosteroid therapy vs. placebo/no therapy [141]. Major limitations to the use of corticosteroids are patient with infections, poorly controlled diabetes mellitus, renal failure, and active gastrointestinal bleeding [142]. The Lille score performed at one week is useful to determine the response to corticosteroid therapy, although recent reports suggest it can be performed at four days as well [143]. Patients with a Lille score >0.45 are determined to be steroid non-responders with a high risk of death; for a score >0.56, treatment should be aborted. For patients with a Lille score between 0.45 and 0.56, guidelines are unclear. It is recommended to continue corticosteroid treatment for one more week and discontinuing if there is no further improvement in a clinical and biochemical profile [142]. Corticosteroid treatment confers a higher risk of fungal infections in the subjects and studies have shown that patients with a higher bacterial DNA load have a higher risk of infections and may benefit from prophylactic antibiotic therapy [144]. Further trials are needed to support concomitant antibiotic therapy to improve outcomes with corticosteroid treatment.

#### 4.3.2. Pentoxifylline

Pentoxifylline is a phosphodiesterase inhibitor that impedes TNF-α activity. Although one study showed benefit of pentoxifylline compared to corticosteroids [145], other studies have failed to show similar benefits [146]. This includes cases where it is used as an adjuvant therapy with corticosteroids, or as salvage therapy for steroid non-responders [147]. It has shown efficacy in reducing mortality in AH patients with hepatorenal syndrome [148], hence there is a likely potential its use in patients with ALD and renal dysfunction. Currently it is used only in centers with a lack of other treatment options, or severe contraindications to corticosteroid therapy [149].

#### 4.3.3. Antioxidants and Micronutrients

Several antioxidants have been studied with respect to ALD, including vitamin E and glutathione. Multiple animal and human models have shown that free radicals and oxidative stress can cause or at least exacerbate liver injury in ALD [150,151]. Vitamin E is a first-line therapy for NASH [152]. However, studies on patients with AH did not show any such benefit [132]. Glutathione is a natural antioxidant, and glutathione prodrugs like *N*-acetylcysteine (NAC) have been used in the management of hepatotoxicity in acetaminophen overdose [153]. Multiple trials have shown that oral antioxidants combined with NAC does not appear to increase survival of AH patients compared with standard medical therapy [154]. However, combination therapy of corticosteroids with NAC was found to improve 1-month survival in patients with severe AH even though it failed to improve 6-month survival [155]. At present, there are no clear benefits on the use of antioxidants for the management of ALD.

Patients with ALD suffer from multiple micronutrient deficiencies, including zinc. Animal models have shown that supplemental zinc protects against ALD by multiple mechanisms including stabilization of gut barrier function, decreasing endotoxemia, decreasing TNF, decreasing oxidative stress and others [156]. Two hundred and twenty milligrams of zinc sulphate supplementation in patients with ALD is being investigated to correct their nutritional deficiency and improve liver disease [157,158].

#### 4.3.4. Interleukin 1 Inhibitors

The pathogenesis of ALD involves upregulation of proinflammatory cytokines, including tumor necrosis factor alpha (TNF-α), interleukin 1 (IL-1) and IL-8 [7,159]. Patients also exhibited neutrophilia, and activation of monocytes and macrophages. IL-1β is a cytokine that signals through IL-1 receptor 1 (IL-1R1), which then leads to an inflammatory cascade [160]. Upregulation of Casp-1 activity and inflammasome activation lead to increased IL-1β as well [160,161]. Mice models have shown that IL-1β signaling is required for the development of alcohol-induced liver injury. A study showed that IL-1 receptor (IL-1R) knockout mice and mice deficient in the inflammasome components casp-1 or Asc had decreased alcohol-induced liver injury, steatosis and inflammation. Treatment of alcohol-fed mice with human recombinant IL-1R antagonist significantly decreased serum ALT, fibrosis markers and liver steatosis and inflammation [106]. Such findings support a potential for using IL-1R1 inhibition in the management of ALD.

#### 4.3.5. TNF-α Inhibitors

TNF-α is a proinflammatory cytokine upregulated in ALD [162]. In mice models, antibodies to TNF-α decreased liver injury. Experimental mice lacking TNF-α receptor did not develop alcohol-induced liver injury [81]. TNF-α inhibition looked like a promising treatment option, unfortunately translation into human research showed limited efficacy. Clinical trials that started the investigation of infliximab and etanercept (TNF-α inhibitors) in the management of severe AH could not be continued due to the higher number of deaths (majorly secondary to infections) in the active treatment arm [163]. Presently TNF-α inhibitors are largely not being investigated for the treatment of ALD.

#### 4.3.6. Granulocyte Colony–Stimulating Factor (G-CSF)

G-CSF is a bone marrow stimulant, which may be useful in treating AH. The mechanism that is involved likely increases the liver regeneration via stimulating engraftment of CD34 stem cells in the liver [2,154]. It could also decrease the incidence of infections by increasing the phagocytic function of neutrophils. Recently, a trial demonstrated improvement in 90-day mortality in patients where G-CSF was combined with pentoxifylline therapy, compared to pentoxifylline alone. This could be attributed to the decreased risk of infections and liver failure [164]. This is a promising avenue for the management of AH [165], and further trials on use with corticosteroids are undergoing in USA.

#### 4.3.7. Fecal Microbiota Transplant (FMT)

Alteration of the fecal microbiome and modulation of the gut-liver axis through healthy donor fecal transplantation (FMT) is an upcoming treatment modality in patients with severe AH [166]. One trial has showed a mortality benefit for FMT compared to treatment with steroids, nutrition and pentoxifylline at the end of one and three months [167]. Large scale studies and randomized controlled trials are warranted for this promising option, although currently there is a FDA warning for potential multi-drug resistant infections from FMT is in place (https://www.fda.gov/vaccines-blood-biologics/safety-availability-biologics/important-safety-alert-regarding-use-fecal-microbiota-transplantation-and-risk-serious-adverse).

### 4.4. Liver Transplant

The end goal for patients with irreversible ALD is liver transplantation [168]. It is a well-accepted management strategy for patients with AC with established sobriety, with alcohol consumption being a factor in approximately 20% of transplants performed in the United States [169]. For patients with severe AH, it is usually not an option as most centers require a 6-month abstinence period before a candidate is evaluated for transplant. However, recent research shows that outcome assessment of the transplant candidates in patients with decompensated ALD is similar to patients with other causes of end-stage liver disease [170,171]. Hence, it is increasingly being used as rescue therapy for cases of severe AH not responding to medical therapy and with life expectancy less than 6 months, who fulfill all other standard criteria for transplantation that includes an evaluation of psychosocial status and alcohol abstinence [172].

## 5. Conclusions

With ALD being a leading cause of reversible morbidity and mortality, significant advances in the understanding of disease pathology have been made. Both direct and indirect biomarkers have the possibility of significantly altering the management of alcohol-induced liver injury. Direct biomarkers are related to alcohol metabolism and have a higher correlation with ALD than indirect biomarkers. Indirect biomarkers can also be elevated by multiple intestinal disorders that would result in false positive cases. While both direct and indirect have an established correlation with ALD, the clinical significance has yet to be established. AST and ALT, which are the primary markers of liver inflammation, are highly unspecific of cause and vary depending on the stage of cirrhosis. The addition of hepatocyte death and inflammatory biomarkers may provide greater insight into inflammation severity and can help in the diagnostic and prognostic aspects of patient management. The direction of AH management will eventually involve the use of these biomarkers.

Research has been aggressively pursued on the management of ALD. Multiple available therapeutic options have not shown higher efficacy, and there are fewer viable therapeutic agents that are still under investigation. Abstinence from alcohol, nutritional support, limited pharmacological options like corticosteroids and liver transplantation are the currently approved management strategies available for treating ALD. More clinical and preclinical studies are needed to investigate the emerging noninvasive biomarkers, such as hepatocyte death markers, proinflammatory cytokine inhibitors and derivations of the fecal microbiota transplant.

## Figures and Tables

**Table 1:** Newly investigated biomarkers of alcoholic hepatitis.

Biomarker	Summary	Methods for Analyzing	Uses
Biomarkers of Liver Cell Death and Regeneration			
Cytokeratin 18 [36,37]	Intracellular intermediate filament protein released during hepatocyte damage	ELISA for M65 and M30 (circulating fragments of cytokeratin-18)	Predicts diagnosis, severity and prognosis of AH
Augmenter of Liver Regeneration (ALR) [38,39]	Protein that promotes liver regeneration, decreased in advanced liver disease	ELISA for serum levels of ALR	Human studies pending; could be used to predict staging of the severity in ALD
Biomarkers of Immune Response			
CD 163 [40,41]	Macrophage receptor protein on Kupffer cells, which are increased in AH	ELISA for plasma concentrations of soluble CD163	Potential to predict severity and prognosis of AH
ST2 Receptor [42,43]	Protein receptors in inflammatory cascade found in hepatocyte inflammation and fibrosis	ELISA for plasma soluble ST2 receptors	Possible therapeutic target; predicts ongoing liver inflammation, staging of ALD severity
TNF- related apoptosis-inducing ligand (TRAIL) [44,45,46]	Inflammatory cytokine released by the Kupffer cells activation, seen in hepatocyte injury	Western blot for serum levels of TRAIL	Potential to predict severity of AH; ongoing research as possible therapeutic target
Immunoglobulins (IgM, IgG, IgA) [47]	Increased in AH	Quantitative serum immunoglobulin tests	Can be used to predict severity of AH;
MicroRNAs (MiR-155, MiR-223) [48,49,50,51]	Noncoding RNAs that regulate expression of their respective target messenger RNA; miR-155 deficiency attenuates chronic alcohol-induced liver injury; MiR-223 is found in neutrophils and increased in AUD	Quantitative PCR for miRNA levels	Can be used to predict severity and prognosis of AH; ongoing research as possible therapeutic target
Biomarkers of Metabolic Changes			
Stearoyl-CoA desaturase 1 (SCD1) [52]	Rate-limiting enzyme that catalyzes the formation of monounsaturated fatty acids and reduced lipid synthesis, influences hepatic inflammation	SCD1 activity can be measured indirectly by the palmitoleic acid to palmitic acid ratio via serum lipid measurements	Ongoing research as possible therapeutic target for early ALD
Magnesium [53,54]	Electrolyte, which is decreased in alcohol use and liver disease	Serum levels	Could predict onset and staging of ALD
Uric acid [55]	Breakdown product of purine metabolism, which is elevated in alcohol use and liver disease	Serum levels	Pro-inflammatory pathological could be used to predict severity in ALD
Biomarkers of Chemical Causes			
Acrolein [56,57]	Toxic metabolite of alcohol metabolism, which accumulates in ALD	Urine tandem mass spectrometry detects the catabolic product of acrolein, 3 hydroxypropyl mercapturic acid (3HMP)	Can be used to predict severity of AH
Resolvins [58]	Lipid mediators that counter-regulate proinflammatory responses, decreased in ALD	ELISA for serum levels of resolvins	Could predict inflammation and severity of AH; ongoing research as possible therapeutic target

**Table 2:** Medical management of alcoholic hepatitis.

Treatment Modality	Summary
Alcohol abstinence	Combination of psychosocial interventions, pharmacological therapy and medical management
Nutritional management	Daily protein intake of 1.2–1.5 g/kg and caloric intake of 35 Kcal/kg, along with micronutrient supplementation
Corticosteroids	Used in severe AH, based on Lille score
Pentoxifylline	Reduces mortality in AH patients with hepatorenal syndrome
Antioxidants and micronutrients	Combination therapy of corticosteroids with NAC may improve survival, no clear cut benefits Micronutrient supplementation encouraged, especially zinc
Interleukin 1 Inhibitors	Human recombinant IL-1R antagonist showed potential in animal models
TNF-α inhibitors	Unsuccessful trials because of high mortality in treatment arm
Granulocyte colony–stimulating factor (G-CSF)	Improvement in 90-day mortality in AH because of decreased risk of infections and liver failure, potential for further use
Fecal microbiota transplant (FMT)	One trial showed benefit, further trials pending
